# Asprosin contributes to nonalcoholic fatty liver disease through regulating lipid accumulation and inflammatory response via AMPK signaling

**DOI:** 10.1002/iid3.947

**Published:** 2023-08-18

**Authors:** Bo Zhang, Jinger Lu, Yuhua Jiang, Yan Feng

**Affiliations:** ^1^ Department of Infectious Disease The Affiliated People's Hospital of Ningbo University Ningbo City Zhejiang Province China; ^2^ Department of Endocrine The Affiliated People's Hospital of Ningbo University Ningbo City Zhejiang Province China; ^3^ Department of Digestive Blood Endocrinology The 75th Group Army Hospital of PLA Dali City Yunnan Province China

**Keywords:** AMPK signaling, Asprosin, inflammatory response, lipid accumulation, nonalcoholic fatty liver disease

## Abstract

**Background:**

Nonalcoholic fatty liver disease (NAFLD) is a primary contributor to liver‐related morbidity and mortality. Asprosin has been reported to be implicated in NAFLD.

**Aims:**

This work is to illuminate the effects of Asprosin on NAFLD and the possible downstream mechanism.

**Materials & Methods:**

The weight of NAFLD mice induced by a high‐fat diet was detected. Quantitative reverse‐transcription polymerase chain reaction (RT‐qPCR) examined serum Asprosin expression. RT‐qPCR and western blot analysis examined Asprosin expression in mice liver tissues. Intraperitoneal glucose tolerance test (IPGTT) and intraperitoneal insulin tolerance test (IPITT) were implemented. Biochemical kits tested liver enzyme levels in mice serum and liver tissues. Hematoxylin and eosin staining evaluated liver histology. Liver weight was also tested and oil red O staining estimated lipid accumulation. RT‐qPCR and western blot analysis analyzed the expression of gluconeogenesis‐, fatty acid biosynthesis‐, fatty acid oxidation‐, and inflammation‐associated factors. Besides, western blot analysis examined the expression of AMP‐activated protein kinase (AMPK)/p38 signaling‐associated factors. In palmitic acid (PA)‐treated mice hepatocytes, RT‐qPCR and western blot analysis examined Asprosin expression. Lipid accumulation, gluconeogenesis, fatty acid biosynthesis, fatty acid oxidation, and inflammation were appraised again.

**Results:**

Asprosin was overexpressed in the serum and liver tissues of NAFLD mice and PA‐treated mice hepatocytes. Asprosin interference reduced mice body and liver weight, improved glucose tolerance and diminished liver injury in vivo. Asprosin knockdown alleviated lipid accumulation and inflammatory infiltration both in vitro and in vivo. Additionally, Asprosin absence activated AMPK/p38 signaling and AMPK inhibitor Compound C reversed the impacts of Asprosin on lipid accumulation and inflammatory response.

**Conclusion:**

Collectively, Asprosin inhibition suppressed lipid accumulation and inflammation to obstruct NAFLD through AMPK/p38 signaling.

## Graphical abstract

Asprosin inhibition obstructed lipid accumulation and inflammatory response in nonalcoholic fatty liver disease (NAFLD) through activating AMP‐activated protein kinase signaling. Therefore, Asprosin might be valued as a potential molecular marker for the treatment of NAFLD.

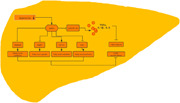

## 1 INTRODUCTION

Nonalcoholic fatty liver disease (NAFLD), a metabolic syndrome due to the deposition of excessive fat in the liver attributed to the causes other than excessive alcohol use, extensively comprises a clinical spectrum from benign liver steatosis to fibrosis and ultimately cirrhosis.^1^ According to statistics, the incidence rate of NAFLD is approximately 25% among adults globally.^2^ Moreover, the morbidity rate has displayed an ascending trend, which increasingly constitutes a grave threat to human health.^3^ As reported, obesity, lipid metabolism disorder, insulin resistance as well as hereditary susceptibility remain dominant risk factors of NAFLD.^4^ In addition, NAFLD is frequently recognized as a pivotal precipitating factor for diversified metabolic diseases, including type 2 diabetes, cardiovascular diseases, liver cirrhosis and cancers.^5^ Growing studies support the crucial roles of lipid and immune pathways in the pathophysiology of NAFLD.^6^, ^7^ Considering that there is no available pharmacological treatment approved by the US Food and Drug Administration and the pathogenesis of NAFLD has not been fully elucidated currently, targeting inflammation and lipid metabolism may be considered as an effective therapeutic intervention for NAFLD.

Asprosin, which was firstly discovered in 2016 by Romere et al.,^8^ is a novel liver‐targeting peptide hormone generated by white adipose tissues involved in the regulation of plasma glucose levels. As reported, the intricate role of Asprosin has been underlined in the central nervous system, peripheral tissues, and organs.^9^ Recent studies have increasingly disclosed that Asprosin is closely implicated in immune and inflammatory diseases, obesity and related cardiovascular diseases are included.^10^, ^11^ Notably, serum Asprosin expression is increased in the patients with NAFLD, which can be used as a valuable biomarker for NAFLD diagnosis.^12^ Moreover, Asprosin expression is positively correlated to the degree of fibrosis in NAFLD.^13^ Nonetheless, the specific role of Asprosin in the progression of NAFLD is indistinct.

AMP‐activated protein kinase (AMPK) is a crucial energy sensor in multiple tissues and organs including the liver, adipose tissue, and muscle.^14^ Emerging evidence has well documented that AMPK can be used as a potential therapeutic target for human cancers and diseases through mediating diverse biological processes including cell growth, cell polarity and migration, autophagy and energy metabolism.^15^, ^16^ Besides, AMPK also serves as a key component that participates in immune‐inflammatory responses.^17^ In particular, activation of AMPK has been supported to hamper the development of NAFLD.^18^

Herin, the current work aims to explore whether Asprosin participates in the process of NAFLD via AMPK signaling.

## 2 MATERIALS AND METHODS

### 2.1 Animal experiments

A total of 20 male C57BL/6J (9 ± 1 weeks old) mice available from Shanghai Sipple‐Bikai Laboratory Animal Co., Ltd were subjected to adaptive feeding for 1 week by being kept in a standard laboratory environment (20°C–26°C, 30%–40% relative humidity) with a 12:12 h light:dark cycle and ad libitum access to food and water. Then four groups were assigned at random: Control group, HFD group, HFD+Lenti‐vector group, and HFD+Lenti‐Asprosin group (*n* = 5). To induce NAFLD, the above mice were fed a high‐fat diet (HFD, D12492, 60% kcal from fat, Research Diet) for 12 weeks continuously.^19^ Mice in the Control group were given a normal chow diet (D12450B, 10% kcal from fat, Research Diet). After fed with HFD, the mice in the HFD+Lenti‐vector group and HFD+Lenti‐Asprosin group were respectively intravenously injected via tail vein with 2 × 10^8^TU/mL lentivirus empty vector (Lenti‐vector) and Asprosin‐silencing lentiviral vector (Lenti‐Asprosin) from RIBOBIO once every 14 days for two times.^20^ Mice's body weight was observed weekly. At the end of the study, the mice were euthanized via intraperitoneally injection of 100 mg/kg sodium pentobarbital. The liver was dissected and liver weight was recorded. The blood samples and liver tissues were collected for further analysis. Animal studies were conducted in conformity with the National Institute of Health Guide for the Care and Use of Laboratory Animals^20^ and reported in compliance with the ARRIVE guidelines.^21^ All animal procedures were approved by the Institutional Animal Care and Use Committee of Jofunhwa Biotechnology (Nanjing) Co., Ltd., (approval number: IACUC‐20210730‐02) in compliance with institutional guidelines for the care and use of animals. All efforts were done to minimize suffering of experimental mice in this research. The experimental flow chart is presented in Figure S1.

### 2.2 Intraperitoneal glucose tolerance test (IPGTT) and intraperitoneal insulin tolerance test (IPITT)

IPGTT and IPITT were conducted 4 weeks after HFD feeding and indicated treatment. Briefly, after fasting for 6 h, the mice were intraperitoneally injected with glucose (2.0 g/kg body weight) or insulin (0.75 U/kg body weight). Blood glucose levels were measured after injection (0, 15, 30, 60, 90, and 120 min) from the tail vein with the application of the Freestyle blood glucose monitoring system (Thera Sense).

### 2.3 Detection of biochemical indexes

Following the centrifugation of blood samples at 1000 *g* for 15 min at 4°C, serum levels of alanine aminotransferase (ALT; cat. no. C009‐2‐1) and aspartate aminotransferase (AST; cat. no. C010‐2‐1), the levels of total cholesterol (TC; cat. no. A111‐1‐1), triglyceride (TG; cat. no. A110‐1‐1) in the serum and liver tissues were respectively analyzed by corresponding commercial kits from Nanjing Jiancheng Bioengineering Institute. In addition, intracellular TG level was also analyzed.

### 2.4 Hematoxylin and eosin (H&E) staining

Mice liver tissues were embedded in 4% paraformaldehyde, before immersion in paraffin and being cut into 5 µm‐thick sections. Following, the processed sections were subjected to staining with H&E solution (Sangon Biotech), and histological evaluation was performed under a light microscope (Keyence).

### 2.5 Cell culture and treatment

Murine hepatocyte cell line AML‐12 supplied by American Type Culture Collection (ATCC) was grown in DMEM/F12 medium (Procell) decorated by 10% fetal bovine serum (Procell), 1% insulin–transferrin–selenium and 1% penicillin–streptomycin at 37°C under 5% CO_2_. AML‐12 cells were exposed to 200 μM palmitate acid (PA; Sigma‐Aldrich) dissolved in bovine serum albumin (Sangon Biotech) for 6 h, 12 h, and 24 h to establish a cellular model of NAFLD.^22^ To verify the role of Asprosin in NAFLD via MAPK signaling, cells were pretreated by 10 μM AMPK inhibitor Compound C (Selleck Chemicals).^23^

### 2.6 Plasmid transfection

Lentiviral vector short hairpin RNA (shRNA) targeting Asprosin (shRNA‐Asprosin) and the corresponding lentivirus empty vector (shRNA‐NC) were transfected into AML‐12 cells with the aid of Attractene reagent (Qiagen GmbH).

### 2.7 Oil red O staining

Mice liver tissues were embedded in 4% paraformaldehyde, before immersion in paraffin and being cut into 5 µm slices. AML‐12 cells were immobilized by 4% paraformaldehyde for 10 min following indicated treatment. Subsequently, the slices and AML‐12 cells were treated with 60% isopropanol for 10 s and incubated in Oil Red O reagent (Sangon Biotech) for 30 min at 4°C. Eventually, the slices and AML‐12 cells were counterstained with hematoxylin following additional washing with 60% isopropanol. The images were captured under a light microscope (Keyence).

### 2.8 Reverse transcription‐quantitative polymerase chain reaction (RT‐qPCR)

Total RNA was prepared from the serum, AML‐12 cells or mice liver tissues adopting Steadypure Universal RNA Extraction Kit (Accurate Biotechnology) separately, followed by the production of cDNA utilizing Evo M‐MLV RT Mix Kit (Accurate Biotechnology). PCR amplification was performed on the CFX Opus 96 Real‐Time PCR system (IMH‐Bio) via the SYBR® Green Pro Taq HS kit (Accurate Biotechnology). The calculation of relative gene expression was achieved by virtue of the 2^−ΔΔCq^ method. GAPDH expression was used for normalization.

### 2.9 Western blot

Total protein was prepared from mice liver tissues and AML‐12 cells adopting radioimmunoprecipitation assay buffer (Beijing Biotyscience Technology Co. Ltd.). Polyvinylidene difluoride membranes were to transfer the protein samples fractionated by 10% sodium dodecyl sulfate‐polyacrylamide gel electrophoresis. Following, the membranes soaked in 5% nonfat milk were successively immunoblotted with primary antibodies overnight at 4°C and goat anti‐rabbit horseradish peroxidase antibody (cat. no. ab205718; 1/2000; Abcam) for 1 h. The blots were visualized by the Omni‐ECLTM Femto Light Chemiluminescence Kit (EpiZyme), and the gray analysis was implemented with Multi Gauge version 3.0 software (Fujifilm). Asprosin (cat. no. FNab09797; 1/1000; Wuhan Fine Biotech Co., Ltd.), 3‐hydroxy‐3‐methylglutaryl‐coA reductase (HMGCR; cat. no. FNab03929; 1/1000; Wuhan Fine Biotech Co., Ltd.), fatty acid binding protein‐1 (FABP1; cat. no. ab171739; 1/1000; Abcam), fatty acid synthase (FAS; cat. no. ab289892; 1/1000; Abcam), carnitine palmitoyltransferase 1A (CPT1A; cat. no. ab234111; 1/1000; Abcam), tumor necrosis factor‐alpha (TNF‐α; cat. no. ab183218; 1/1000; Abcam), interleukin‐1beta (IL‐1β; cat. no. ab254360; 1/1000; Abcam), interleukin‐6 (IL‐6; cat. no. ab290735; 1/1000; Abcam), p65 (cat. no. ab32536; 1/1000; Abcam), phosphorylated (p)‐p65 (cat. no. ab76302; 1/1000; Abcam), and β‐actin (cat. no. ab8227; 1/1000) primary antibodies were used here.

### 2.10 Statistical analyses

All statistical analyses were executed using GraphPad Prism 8 software (GraphPad Software, Inc.), and continuous variables were given as mean ± standard deviation (SD) from three independent experiments. Comparisons between two groups were done by Student's *t*‐test, and differences among other groups were estimated by one‐way analysis of variance (ANOVA) as well as Tukey's post hoc test. *p* less than .05 was the threshold of significance.

## 3 RESULTS

### 3.1 Asprosin expression is increased in the serum and liver tissues of NAFLD mice

**Figure 1:**
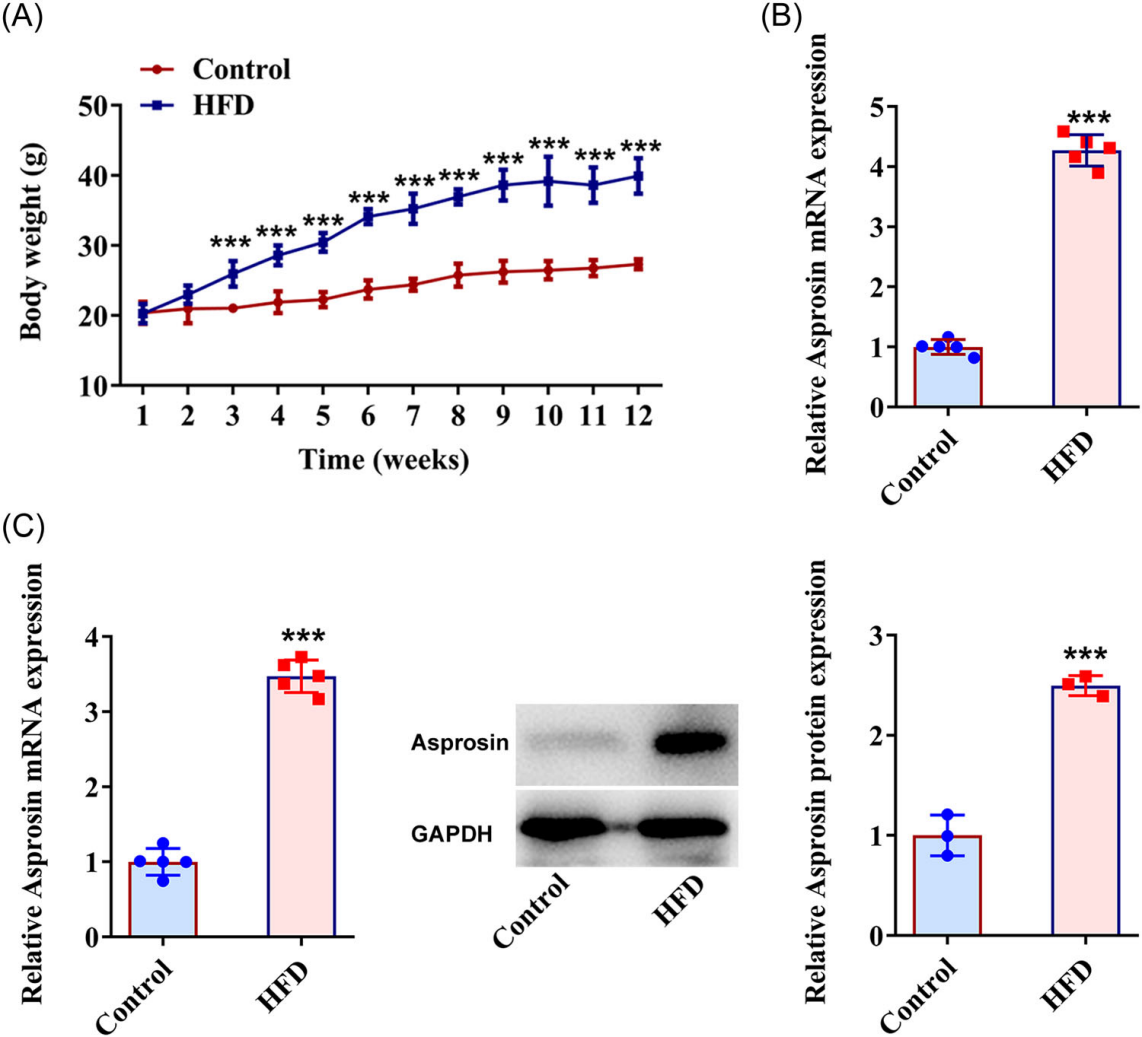
Asprosin expression is increased in the serum and liver tissues of NAFLD mice (*n* = 5/group). (A) Examination of mice body weight. (B) RT‐qPCR tested Asprosin expression in the mice serum. (C) RT‐qPCR and western blot tested Asprosin expression in mice liver tissues. Data are presented as mean ± SD. ***p < .001 versus Control. GAPDH, glyceraldehyde‐3‐phosphate dehydrogenase; HFD, high‐fat diet; NAFLD, nonalcoholic fatty liver disease; RT‐qPCR, quantitative reverse‐transcription polymerase chain reaction; SD, standard deviation.

To verify the successful establishment of NAFLD mice, mice body weight was estimated during the experiment period. As depicted in Figure 1A, the body weight of the mice was on a gradual upward trend in the HFD group relative to the Control group. Through RT‐qPCR analysis, it was discovered that Asprosin expression was prominently raised in the mice serum in the HFD group by contrast with the Control group (Figure 1B). Further, RT‐qPCR and western blot analyzed that Asprosin also exhibited relatively higher expression in the mice liver tissues in the HFD group than the Control group (Figure 1C). Accordingly, Asprosin was overexpressed in the HFD‐induced NAFLD mice model.

### 3.2 Asprosin interference reduces mice body weight, improves glucose tolerance and insulin sensitivity in HFD‐induced NAFLD mice model

**Figure 2:**
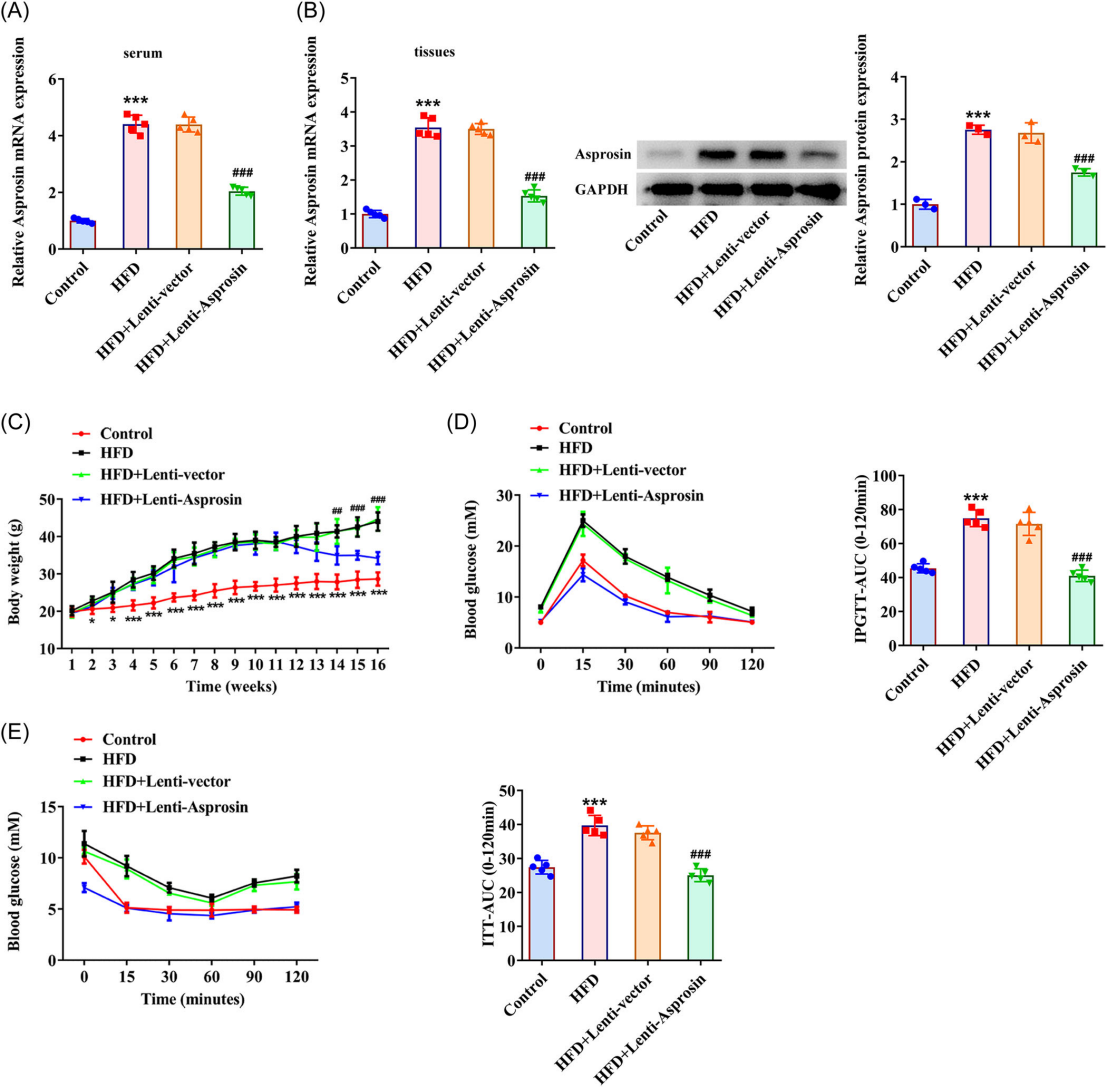
Asprosin interference reduces mice body weight, improves glucose tolerance, and insulin sensitivity in HFD‐induced NAFLD mice model (*n* = 5/group). (A) RT‐qPCR tested Asprosin expression in the mice serum following transduction of Lenti‐Asprosin. (B) RT‐qPCR and western blot tested Asprosin expression in mice liver tissues following transduction of Lenti‐Asprosin. (C) Examination of mice body weight. (D) IPGTT and (E) IPITT tested glucose tolerance and insulin tolerance. Data are presented as mean ± SD. *p < .05, ***p < .001 versus Control. ^##^ *p* < .01, ^###^ *p* < .001 versus HFD+Lenti‐vector. AUC, area under curve; GAPDH, glyceraldehyde‐3‐phosphate dehydrogenase; HFD, high‐fat diet; IPGTT, intraperitoneal glucose tolerance test; ITT; insulin tolerance test; NAFLD, nonalcoholic fatty liver disease; RT‐qPCR, quantitative reverse‐transcription polymerase chain reaction; SD, standard deviation.

RT‐qPCR and western blot analysis manifested that after transduction of Lenti‐ Asprosin, Asprosin expression was notably depleted in the serum and liver tissues of HFD‐induced NAFLD mice (Figure 2A,B). Following silencing of Asprosin, mice body weight was assessed again and the results illuminated that HFD treatment markedly elevated mice body weight, which was then declined from the 11th week by interference with Asprosin (Figure 2C). In addition, as Figure 2D displayed, HFD treatment resulted in the increase in the blood glucose levels and area under curve (AUC) during IPGTT and IPITT. However, lower blood glucose levels and AUC were noticed in HFD‐treated mice when Asprosin was downregulated (Figure 2E), suggesting that Asprosin depletion improved glucose tolerance and insulin sensitivity. All these findings implied that Asprosin inhibition might reduce HFD‐stimulated increase on mice body weight and decrease in glucose tolerance and insulin sensitivity.

### 3.3 Asprosin absence alleviates hepatic function injury and pathological damage of liver tissues of HFD‐induced NAFLD mice

**Figure 3:**
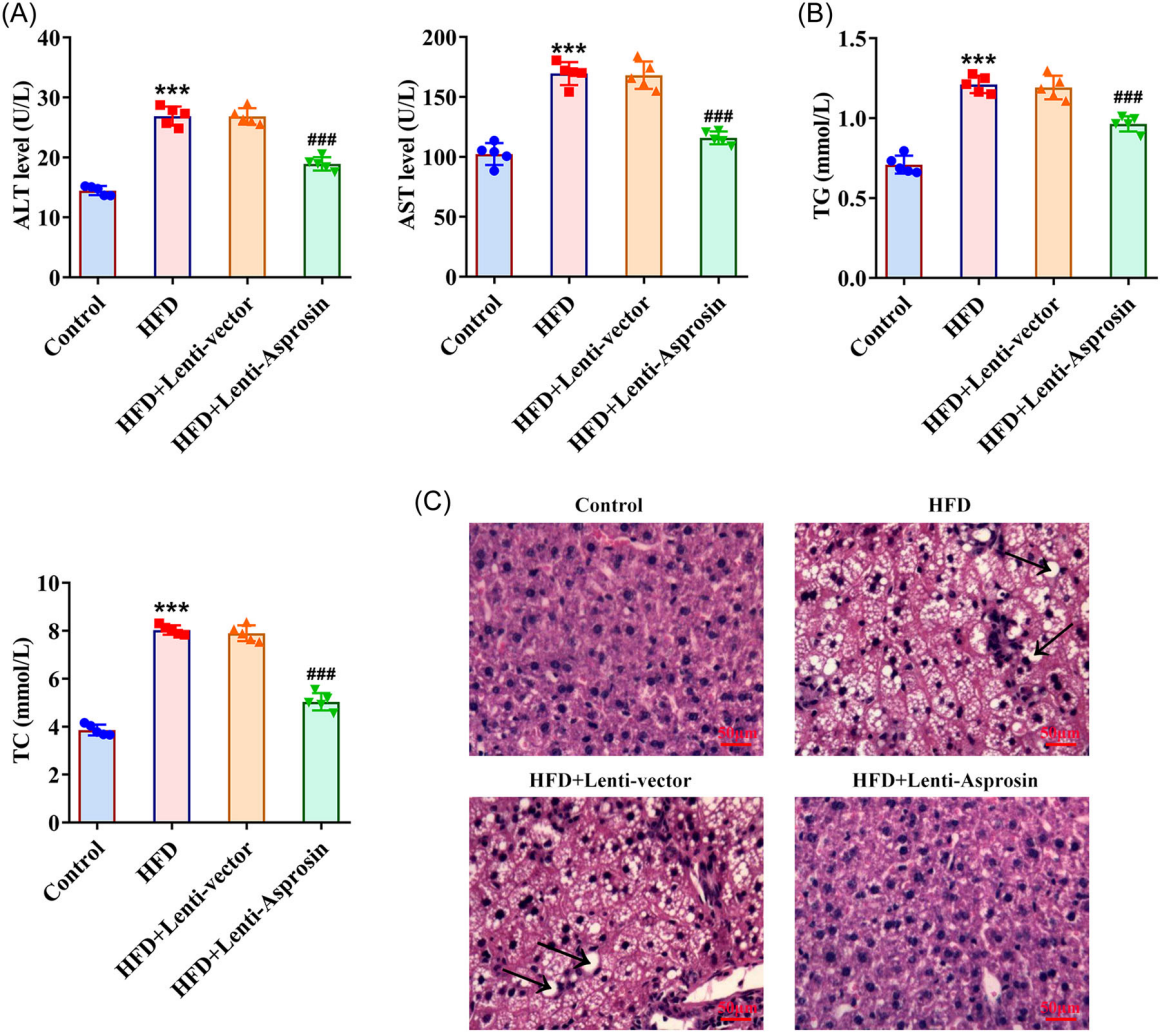
Asprosin absence alleviates hepatic function injury and pathological damage of liver tissues of HFD‐induced NAFLD mice (*n* = 5/group). Related kits tested (A) ALT, AST, (B) TG, and TC levels in the mice serum. (C) H&E staining evaluated pathological alternations of mice liver tissues (×400). Data are presented as mean ± SD. ***p < .001 versus Control. ^###^ *p* < .001 versus HFD+Lenti‐vector. ALT, alanine aminotransferase; AST, aspartate aminotransferase; H&E, hematoxylin and eosin; HFD, high‐fat diet; NAFLD, nonalcoholic fatty liver disease; SD, standard deviation; TC, total cholesterol; TG, triglyceride.

To evaluate the effects of Asprosin on a liver injury during the process of NAFLD, the levels of liver enzymes were examined, and the results presented that the remarkably ascending ALT, AST, TG, and TC levels in the mice serum induced by HFD were all declined by Asprosin deficiency (Figure 3A,B). Besides, it was observed through H&E staining that HFD‐elicited severe pathological damage to mice liver tissues was diminished after Asprosin was silenced (Figure 3C). Overall, inhibition of Asprosin might protect against HFD‐stimulated hepatic dysfunction and damage in NAFLD mice.

### 3.4 Asprosin absence mitigates lipid accumulation in HFD‐stimulated NAFLD mice

**Figure 4:**
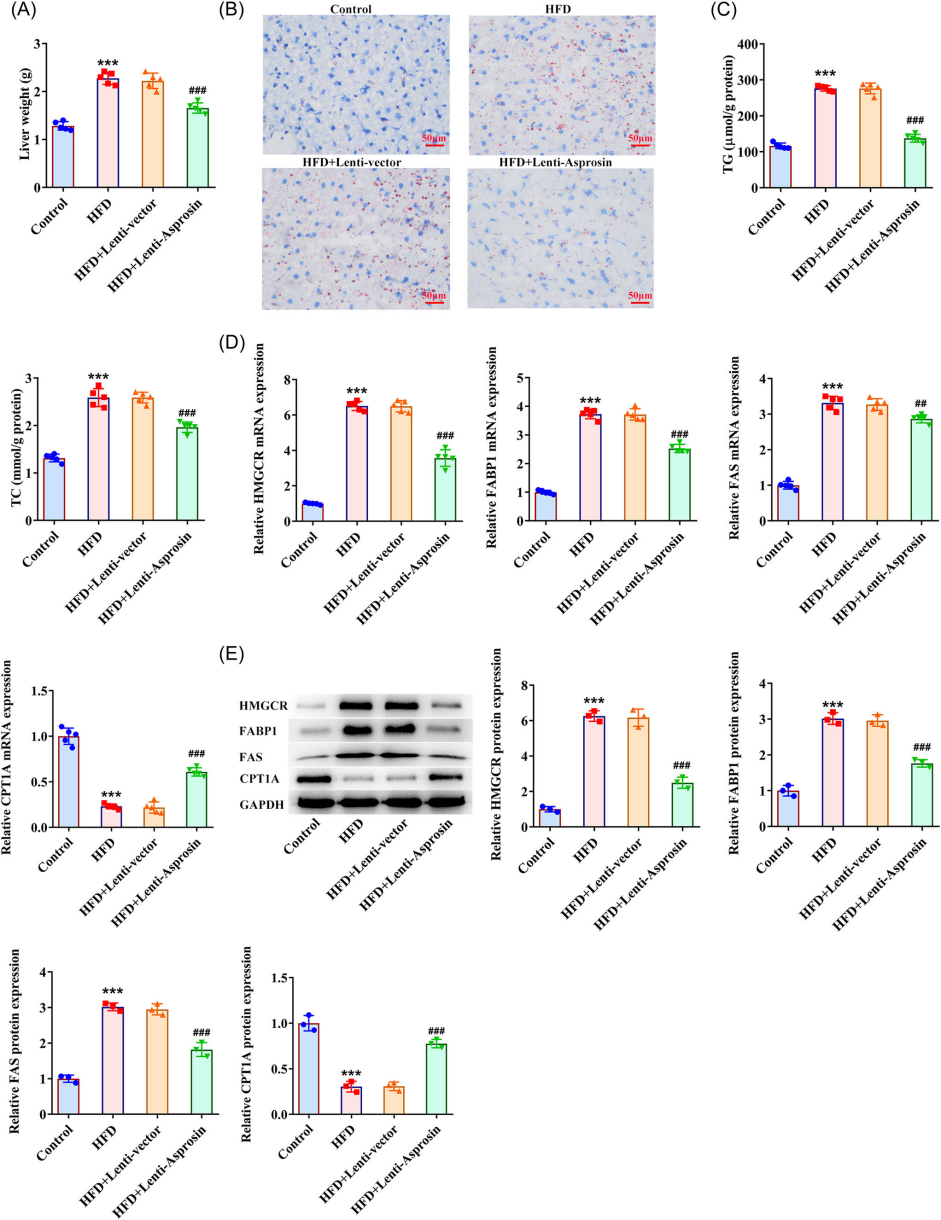
Asprosin absence mitigates lipid accumulation in HFD‐stimulated NAFLD mice (*n* = 5/group). (A) Examination of mice liver weight. (B) Oil red O staining estimated lipid deposition (×400). (C) Related kits tested TG and TC levels in mice liver tissues. (D) RT‐qPCR and (E) western blot tested the expression of gluconeogenesis‐, fatty acid biosynthesis‐ and fatty acid oxidation‐associated factors. Data are presented as mean ± SD. ***p < .001 versus Control. ^##^ *p* < .01, ^###^ *p* < .001 versus HFD+Lenti‐vector. CPT1A, carnitine palmitoyltransferase 1A; FABP1, fatty acid binding protein‐1; FAS, fatty acid synthase; GAPDH, glyceraldehyde‐3‐phosphate dehydrogenase; HFD, high‐fat diet; HMGCR, 3‐hydroxy‐3‐methylglutaryl‐coA reductase; NAFLD, nonalcoholic fatty liver disease; RT‐qPCR, quantitative reverse‐transcription polymerase chain reaction; SD, standard deviation; TC, total cholesterol; TG, triglyceride.

In addition, the increase in mice liver weight following HFD treatment was also abrogated by knockdown of Asprosin (Figure 4A). The results of Oil red O staining elucidated that the obvious hepatic lipid deposition in HFD‐induced NAFLD mice was notably eased following interference with Asprosin (Figure 4B). Also, Asprosin downregulation lessened the elevated TG and TC levels in the liver tissues of NAFLD mice treated by HFD (Figure 4C). Concurrently, the expression of gluconeogenesis‐, fatty acid biosynthesis‐ and fatty acid oxidation‐associated factors were examined with RT‐qPCR and western blot and it was noticed that HMGCR, FABP1, FAS expression was augmented while CPT1A expression was cut down in the liver tissues of NAFLD mice upon exposure to HFD treatment, which was all restored when Asprosin was downregulated (Figure 4D,E). In summary, Asprosin knockdown suppressed HFD‐triggered lipid accumulation in NAFLD mice.

### 3.5 Asprosin insufficiency ameliorates inflammatory infiltration in HFD‐stimulated NAFLD mice

**Figure 5:**
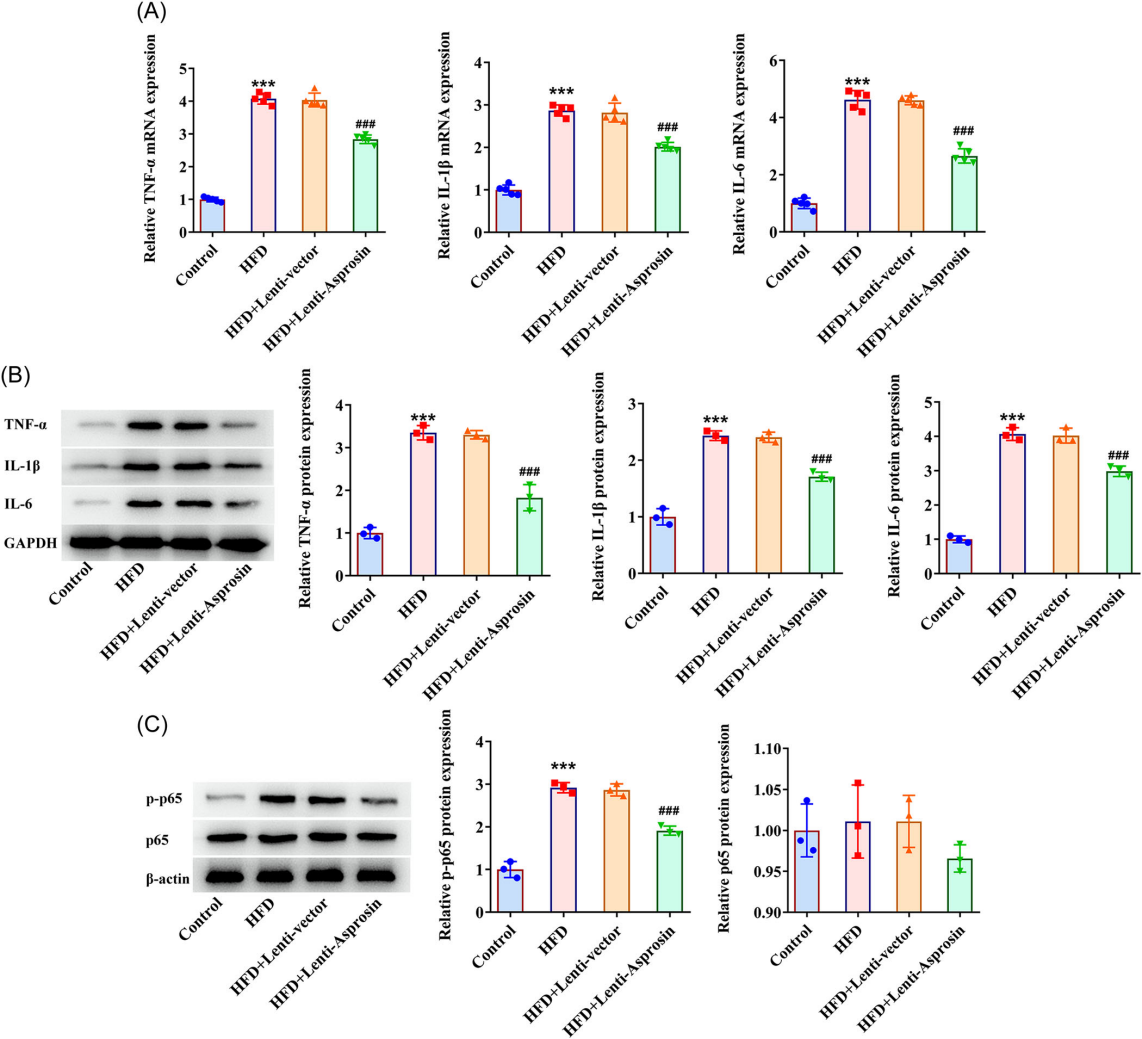
Asprosin insufficiency ameliorates inflammatory infiltration in HFD‐stimulated NAFLD mice (*n* = 5/group). (A) RT‐qPCR and (B) western blot tested the expression of inflammatory enzymes. (C) Western blot tested the expression of p‐p65 and p65. Data are presented as mean ± SD. ***p < .001 versus Control. ^###^ *p* < .001 versus HFD+Lenti‐vector. GAPDH, glyceraldehyde‐3‐phosphate dehydrogenase; HFD, high‐fat diet; IL‐1β, interleukin‐1beta. IL‐6, interleukin‐6; NAFLD, nonalcoholic fatty liver disease; p‐p65, phosphorylated‐p65; RT‐qPCR, quantitative reverse‐transcription polymerase chain reaction; SD, standard deviation; TNF‐α, Tumor necrosis factor‐alpha.

Inflammation has been recognized as a pivotal driver of NAFLD development. The data from RT‐qPCR and western blot delineated that the aggrandized expression of inflammatory cytokines including TNF‐α, IL‐1β, and IL‐6 in the liver tissues of NAFLD mice caused by HFD treatment were all declined due to deficiency of Asprosin (Figure 5A,B). Similarly, silencing of Asprosin led to the reduction on the raised p‐p65 expression in the liver tissues of HFD‐stimulated NAFLD mice (Figure 5C). To be concluded, Asprosin depletion could act as an inflammatory suppressor in HFD‐stimulated NAFLD mice.

### 3.6 Asprosin insufficiency activates AMPK‐p38 signaling in HFD‐stimulated NAFLD mice

**Figure 6:**
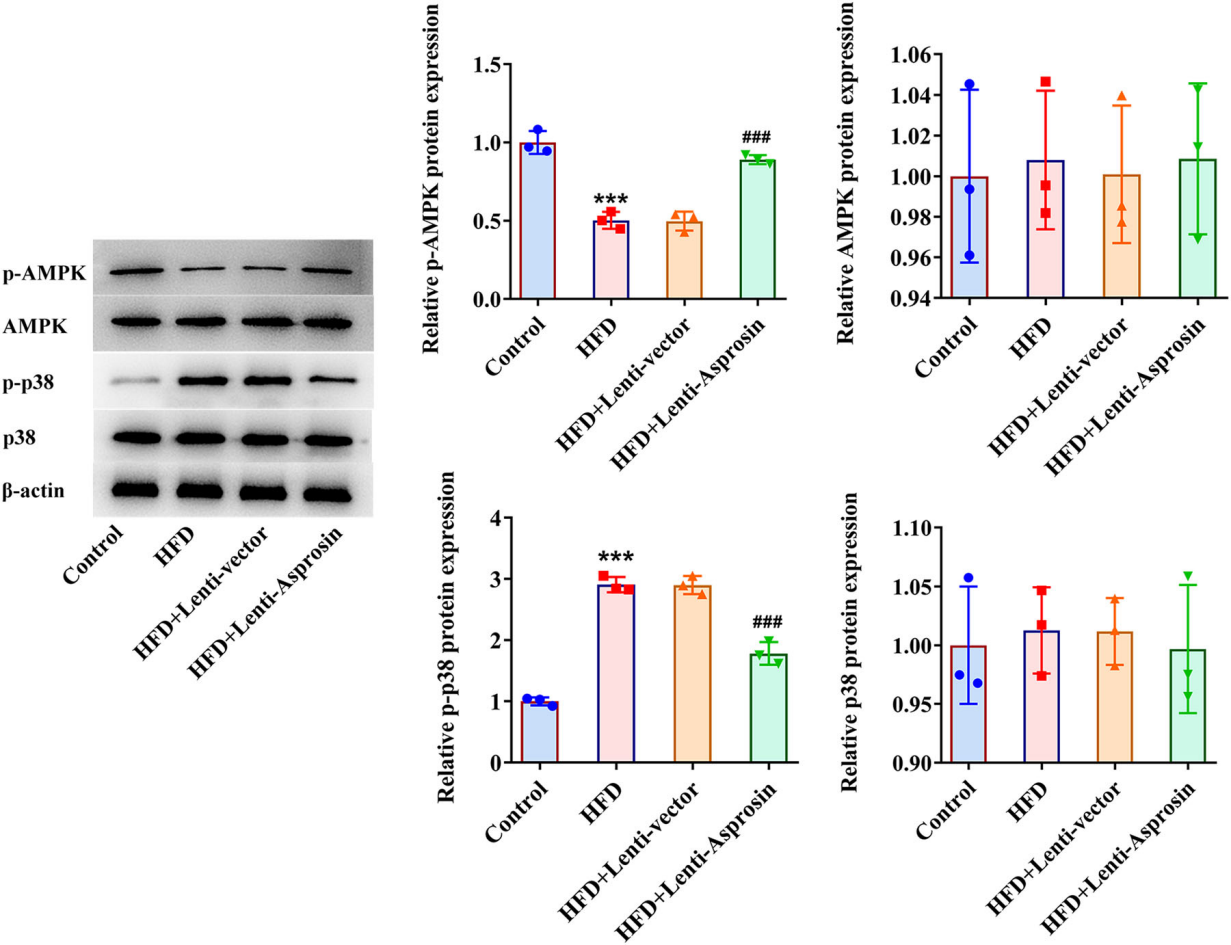
Asprosin insufficiency activates AMPK‐p38 signaling in HFD‐stimulated NAFLD mice (*n* = 5/group). Western blot analyzed the expression of AMPK‐p38 signaling‐associated proteins. Data are presented as mean ± SD. ***p < .001 versus Control. ^###^ *p* < .001 versus HFD+Lenti‐vector. AMPK, AMP‐activated protein kinase; HFD, high‐fat diet; NAFLD, nonalcoholic fatty liver disease; SD, standard deviation.

Intriguingly, HFD treatment lowered p‐AMPK expression while p‐p38 expression. When Asprosin was downregulated, p‐AMPK expression was elevated and p‐p38 expression was depleted in HFD‐treated NAFLD mice (Figure 6), suggesting that inhibition of Asprosin might be viewed as an activator of AMPK‐p38 signaling in HFD‐stimulated NAFLD mice.

### 3.7 Asprosin interference suppresses lipid deposition in PA‐exposed AML‐12 cells

**Figure 7:**
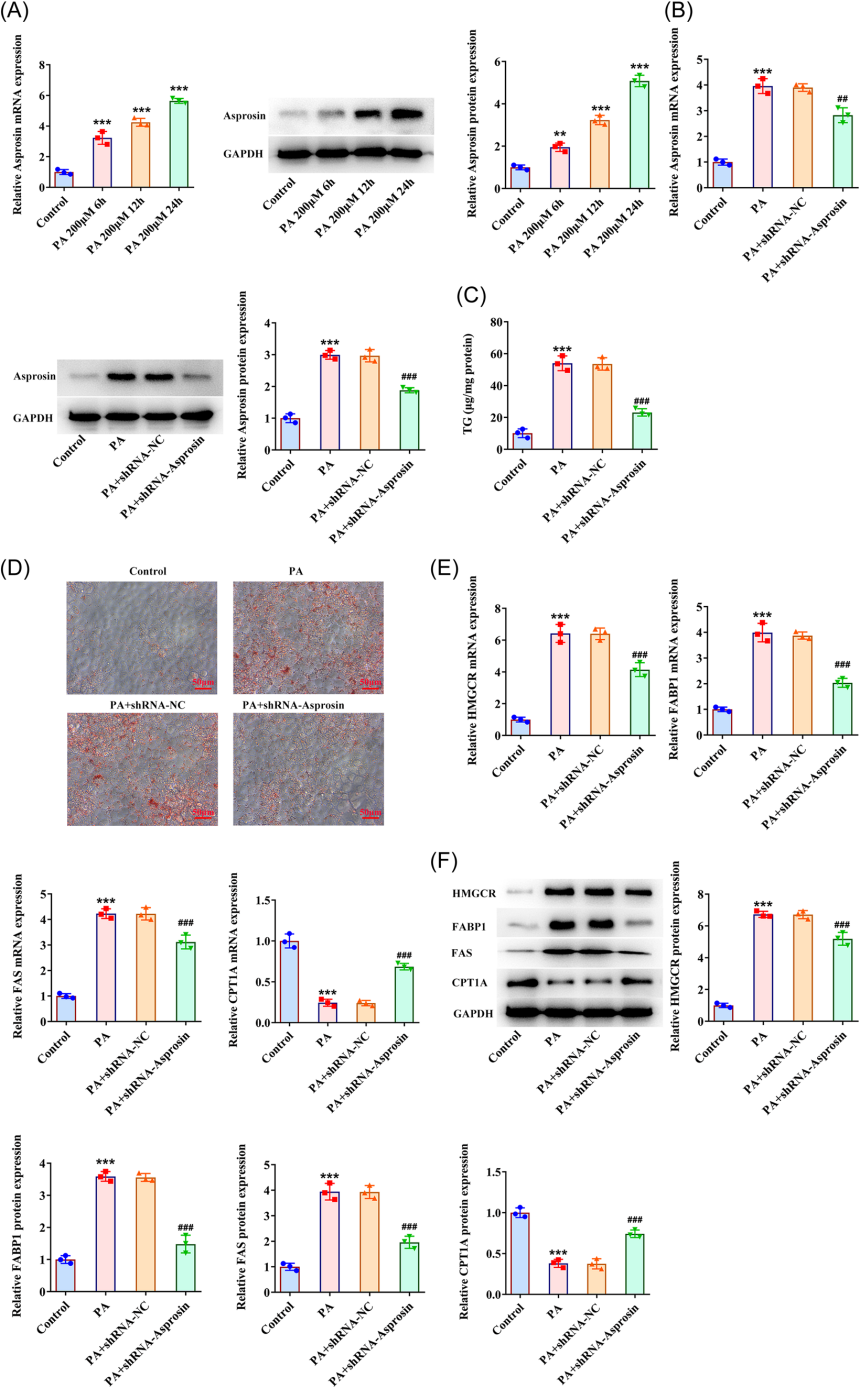
Asprosin interference suppresses lipid deposition in PA‐exposed AML‐12 cells. (A) RT‐qPCR and western blot tested Asprosin expression in AML‐12 cells challenged with PA. (B) RT‐qPCR and western blot tested Asprosin expression in PA‐exposed AML‐12 cells following transduction of shRNA‐Asprosin. (C) Related kits tested TG level. (D) Oil red O staining estimated lipid deposition (×400). (E) RT‐qPCR and (F) western blot tested the expression of gluconeogenesis‐, fatty acid biosynthesis‐, and fatty acid oxidation‐associated factors. Data are presented as mean ± SD. **p < .01, ***p < .001 versus Control. ^##^ *p* < .01, ^###^ *p* < .001 versus PA+shRNA‐NC. GAPDH, glyceraldehyde‐3‐phosphate dehydrogenase; CPT1A, carnitine palmitoyltransferase 1A; FABP1, fatty acid binding protein‐1; FAS, fatty acid synthase; HMGCR, 3‐hydroxy‐3‐methylglutaryl‐coA reductase; PA, palmitic acid; qRT‐PCR, quantitative reverse‐transcription polymerase chain reaction; SD, standard deviation; shRNA, short hairpin RNA; TG, triglyceride.

To comprehensively clarify the role of Asprosin in NAFLD, Asprosin expression was further tested in PA‐treated AML‐12 cells and Asprosin was found to be overexpressed with the increasing time of PA treatment. Thereafter, 24 h treatment of PA was selected for the ensuing experiments as Asprosin expression was the highest when treated by PA for 24 h (Figure 7A). After transfection of shRNA‐Asprosin, the raised Asprosin expression in PA‐challenged AML‐12 cells was noticed to be distinctly depleted (Figure 7B). As expected, PA‐stimulated TG level was conspicuously reduced after Asprosin was silenced (Figure 7C). Further, the obvious lipid accumulation brought about by PA was lightened when Asprosin was downregulated (Figure 7D). Also, Asprosin absence downregulated HMGCR, FABP1, FAS expression whereas upregulated CPT1A expression in PA‐treated AML‐12 cells (Figure 7E,F). All in all, Asprosin deficiency hindered PA‐stimulated lipid deposition in AML‐12 cells.

### 3.8 Asprosin interference eases inflammatory response in PA‐challenged AML‐12 cells

**Figure 8:**
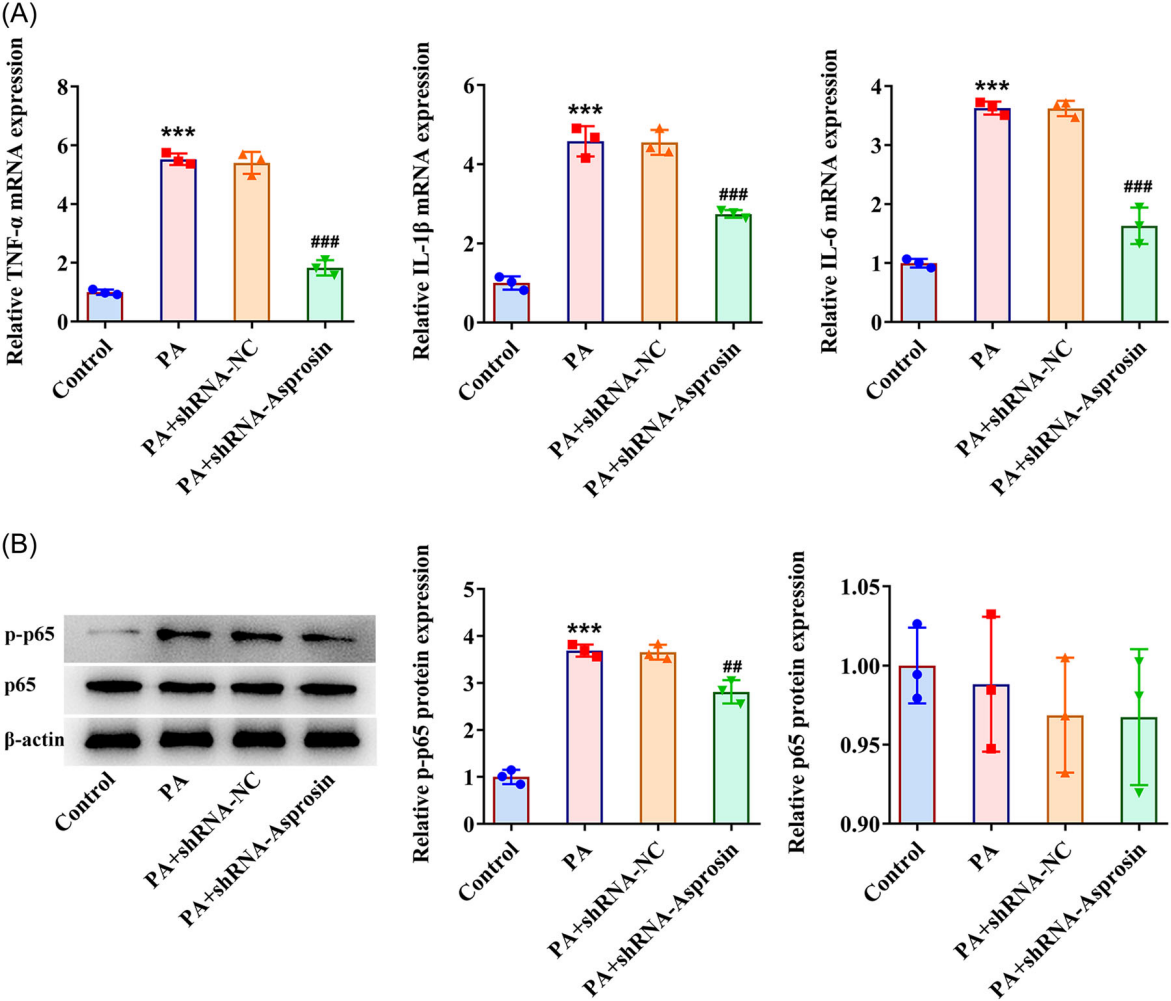
Asprosin interference eases inflammatory response in PA‐challenged AML‐12 cells. (A) RT‐qPCR tested the expression of inflammatory enzymes. (B) Western blot tested the expression of p‐p65 and p65. Data are presented as mean ± SD. ***p < .001 versus Control. ^##^ *p* < .01, ^###^ *p* < .001 versus PA+shRNA‐NC. PA, palmitic acid; p‐p65, phosphorylated‐p65; qRT‐PCR, quantitative reverse‐transcription polymerase chain reaction; SD, standard deviation.

At the same time, PA was discovered to increase TNF‐α, IL‐1β, IL‐6, and p‐p65 expression in AML‐12 cells. Following transfection of Asprosin interference plasmid, TNF‐α, IL‐1β, IL‐6, and p‐p65 expression were all diminished in AML‐12 cells challenged with PA (Figure 8A,B), which implied that Asprosin silencing might exert anti‐inflammatory activities on PA‐exposed AML‐12 cells.

### 3.9 Asprosin absence activates AMPK‐p38 signaling to diminish PA‐evoked lipid aggregation and inflammatory response in AML‐12 cells

**Figure 9:**
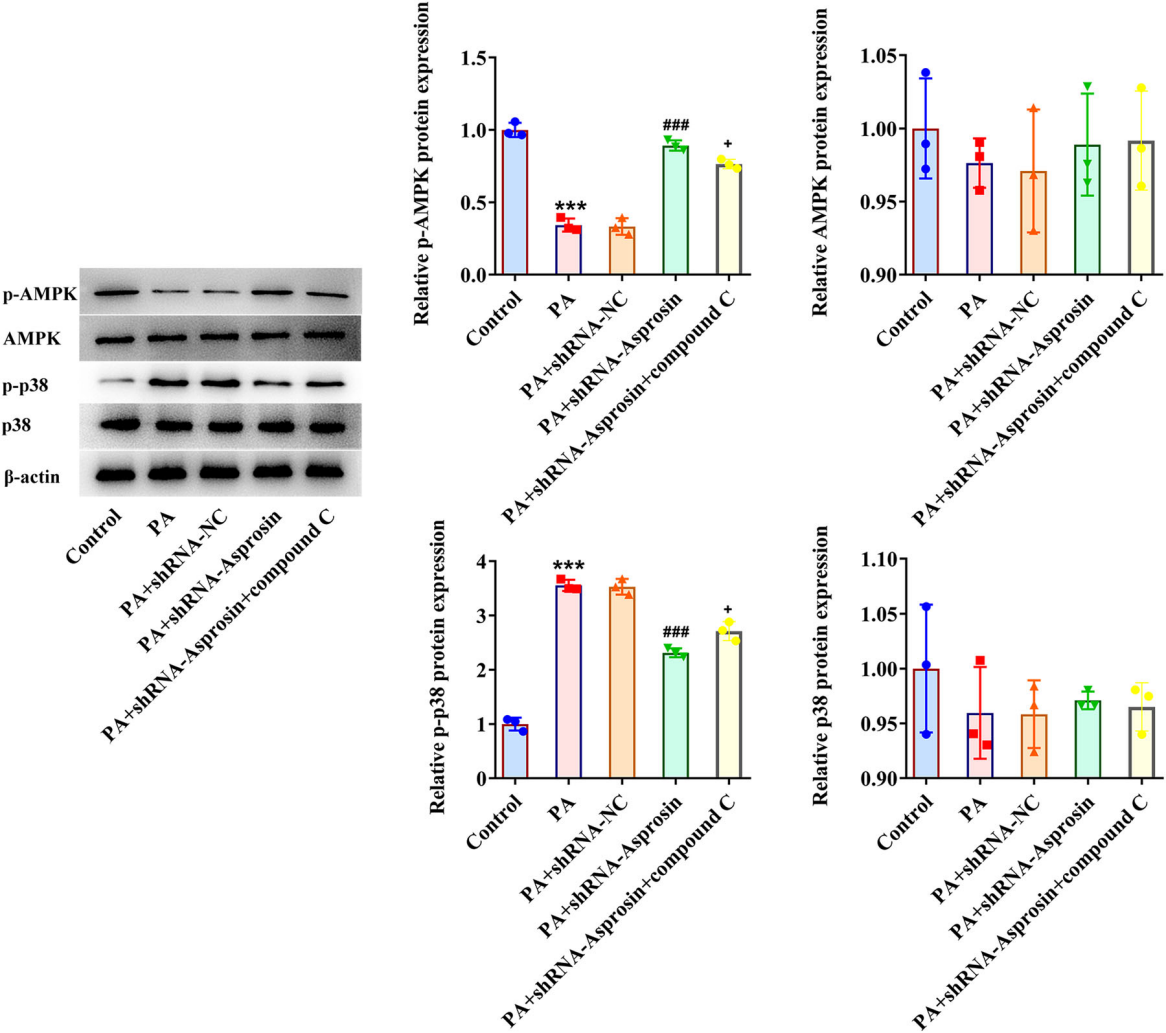
AMPK inhibitor reverses the impacts of Asprosin inhibition on AMPK‐p38 signaling in AML‐12 cells exposed to PA. (A–C) Western blot analyzed the expression of AMPK‐p38 signaling‐associated proteins. Data are presented as mean ± SD. ***p < .001 versus Control. ^###^ *p* < .001 versus PA+shRNA‐NC. ^+^ *p* < .05 versus PA+shRNA‐Asprosin. AMPK, AMP‐activated protein kinase; PA, palmitic acid; qRT‐PCR, quantitative reverse‐transcription polymerase chain reaction; SD, standard deviation; shRNA, short hairpin RNA.

**Figure 10:**
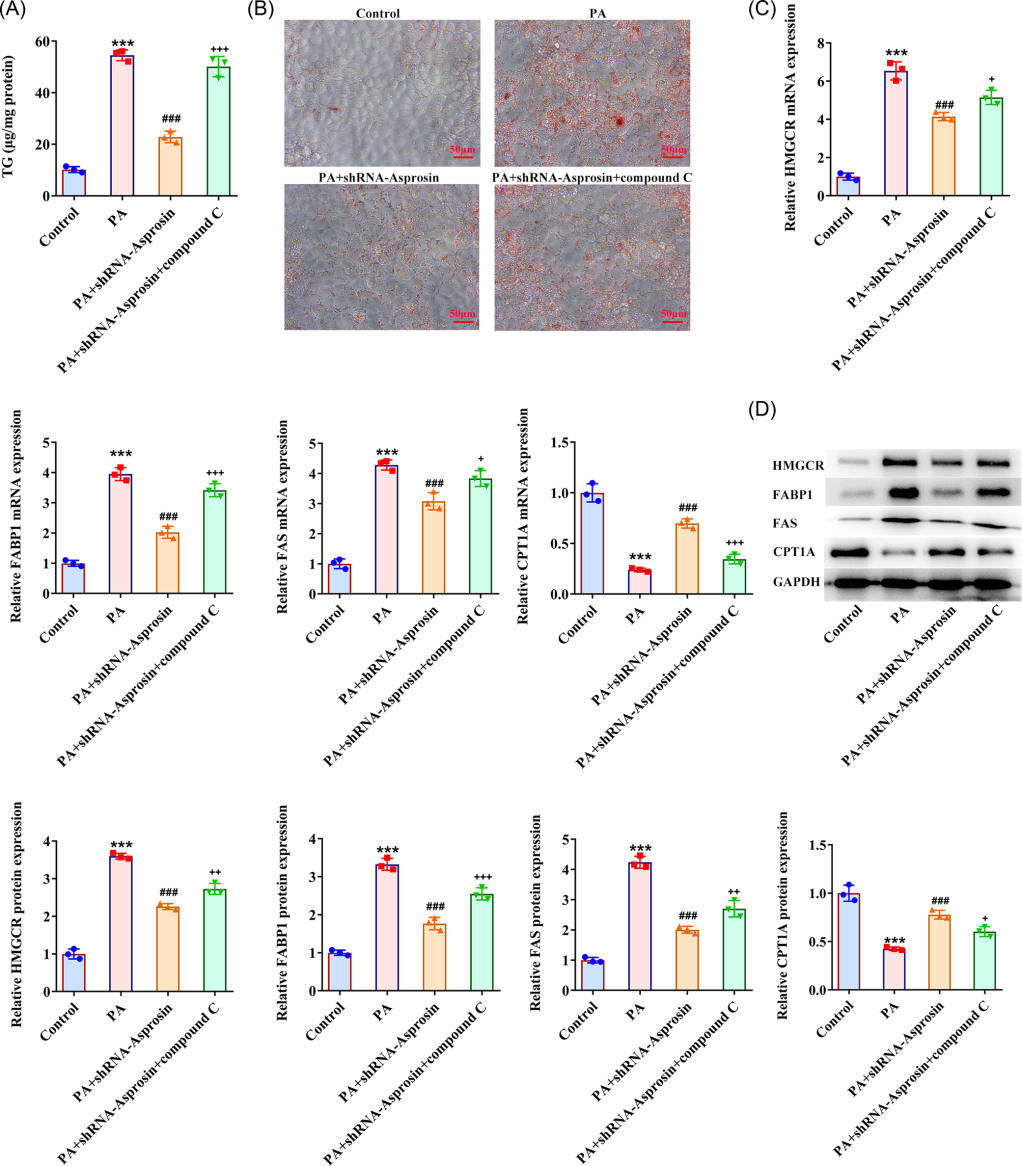
Asprosin absence activates AMPK‐p38 signaling to diminish PA‐evoked lipid aggregation in AML‐12 cells. (A) Related kits tested TG level. (B) Oil red O staining estimated lipid deposition (×400). (C) RT‐qPCR and (D) western blot tested the expression of gluconeogenesis‐, fatty acid biosynthesis‐, and fatty acid oxidation‐associated factors. Data are presented as mean ± SD. ***p < .001 versus Control. ^###^ *p* < .001 versus PA+shRNA‐NC. ^+^ *p* < .05, ^++^ *p* < .01, ^+++^ *p* < .001 versus PA+shRNA‐Asprosin. AMPK, AMP‐activated protein kinase; CPT1A, carnitine palmitoyltransferase 1A; FABP1, fatty acid binding protein‐1; FAS, fatty acid synthase; GAPDH, glyceraldehyde‐3‐phosphate dehydrogenase; HMGCR, 3‐hydroxy‐3‐methylglutaryl‐coA reductase; PA, palmitic acid; qRT‐PCR, quantitative reverse‐transcription polymerase chain reaction; SD, standard deviation; shRNA, short hairpin RNA; TG, triglyceride.

**Figure 11:**
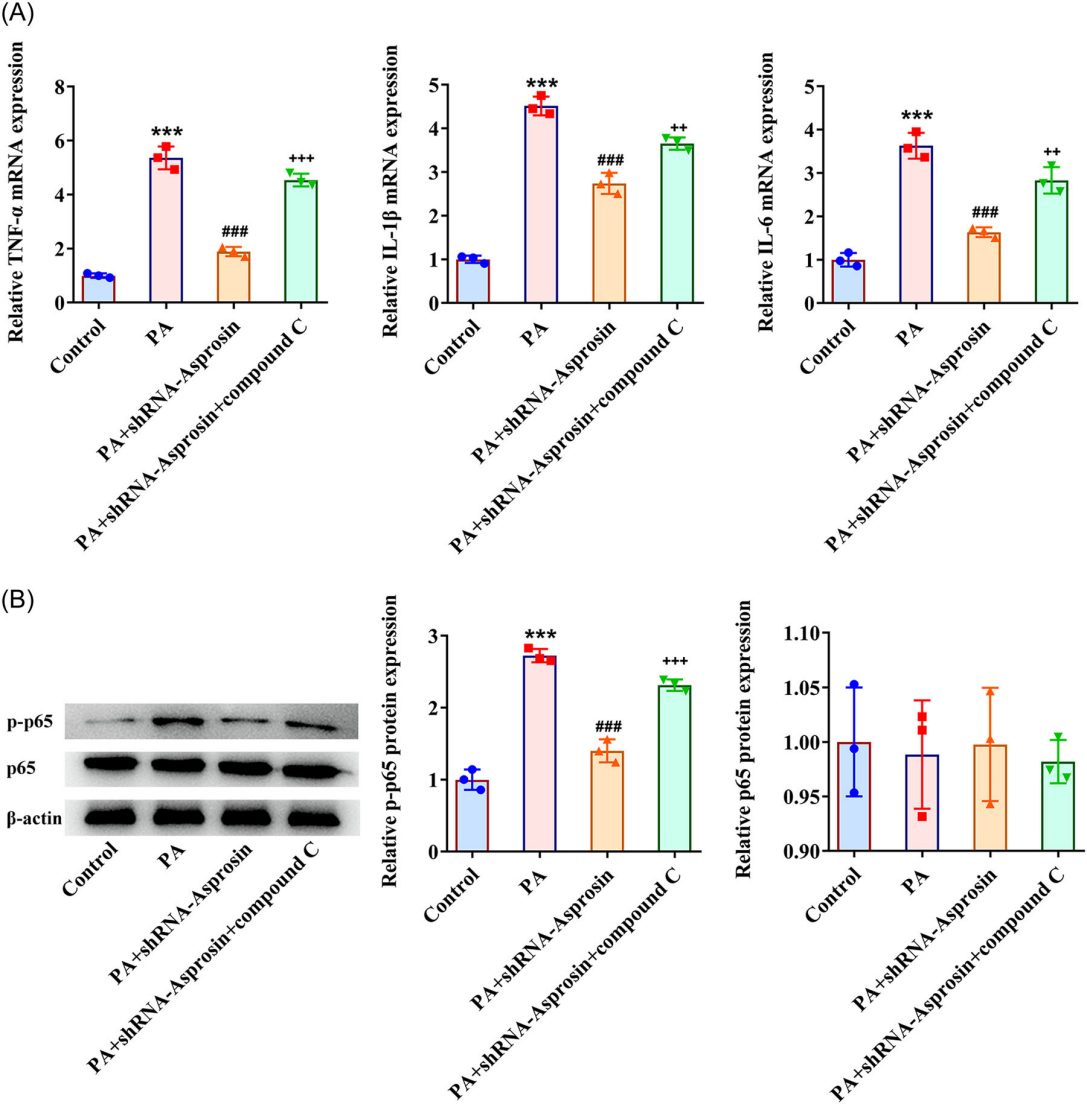
Asprosin absence activates AMPK‐p38 signaling to relieve PA‐elicited inflammatory response in AML‐12 cells. (A) RT‐qPCR tested the expression of inflammatory enzymes. (B) Western blot tested the expression of p‐p65 and p65. Data are presented as mean ± SD. ***p < .001 versus Control. ^###^ *p* < .001 versus PA+shRNA‐NC. ^++^ *p* < .01, ^+++^ *p* < .001 versus PA+shRNA‐Asprosin. AMPK, AMP‐activated protein kinase; IL‐1β, interleukin‐1beta; IL‐6, interleukin‐6; PA, palmitic acid; p‐p65, phosphorylated‐p65; qRT‐PCR, quantitative reverse‐transcription polymerase chain reaction; SD, standard deviation; shRNA, short hairpin RNA; TNF‐α, tumor necrosis factor‐alpha.

To corroborate that Asprosin participated in lipid accumulation and inflammatory response during NAFLD through modulating AMPK‐p38 signaling, AMPK inhibitor Compound C was employed. As expected, Asprosin reduction augmented p‐AMPK expression and suppressed p‐p38 expression in AML‐12 cells upon exposure to PA, the impacts of which were partially abolished by the addition of Compound C (Figure 9A,C). Notably, the depleted TG level in PA‐challenged AML‐12 cells imposed by Asprosin shortage was enhanced again following inactivation of AMPK (Figure 10A). In the same way, Asprosin interference diminished PA‐stimulated lipid accumulation in AML‐12 cells, which was then further restored by Compound C (Figure 10B). Additionally, Compound C partially reversed the descending HMGCR, FABP1, FAS expression, and ascending CPT1A expression on account of Asprosin absence in PA‐treated AML‐12 cells (Figure 10C,D). What's more, Compound C abrogated the inhibitory role of Asprosin depletion in TNF‐α, IL‐1β, IL‐6, and p‐p65 expression in AML‐12 cells treated by PA (Figure 11A,B). Collectively, AMPK inhibitor countervailed the impacts of Asprosin interference on lipid deposition and inflammatory response in PA‐stimulated AML‐12 cells.

## 4 DISCUSSION

NAFLD is currently recognized as a metabolic stress liver injury featured by hepatocyte steatosis and increased lipid deposition.^24^, ^25^ A great deal of research has documented that excess fat intake presents a high risk of NAFLD.^26^, ^27^ Thence, in this study, the mice were induced by HFD to construct an in vivo model of NAFLD, and the experimental results showed that the body weight of the mice was on a gradual upward trend following HFD treatment. Besides, long‐term exposure to PA, a kind of saturated free fatty acid (FFA), has been extensively reported to induce hepatocyte damage for in vitro experiments.^28^ Accordingly, PA was utilized to establish an in vitro model of NAFLD in AML‐12 cells here.

Adipokines have been viewed as regulators of immune responses, and adipokines that have immunometabolic activity are involved in the pathogenesis and development of NAFLD.^29^, ^30^ Aa a novel glucogenic adipokine, Asprosin has been supported to play an important regulatory role in the glucose metabolism of the liver.^31^ It is worth noticing that serum Asprosin expression is increased in patients with NAFLD, which may be implicated in the pathogenesis of NAFLD.^12^, ^32^ Moreover, Asprosin expression has a close association with the degree of fibrosis in NAFLD.^13^ Consistently, Asprosin expression was discovered to be elevated in the serum and liver tissues of HFD‐induced NAFLD mice and PA‐challenged AML‐12 cells. Following the silencing of Asprosin, mice body weight was prominently reduced. In addition, HFD treatment resulted in an increase in the blood glucose levels and AUC during IPGTT and IPITT, which were then declined by Asprosin absence, revealing that Asprosin depletion improved glucose tolerance and insulin sensitivity in NAFLD. ALT and AST levels are important criterion for the evaluation of liver injury^33^ whereas TC and TG are lipid‐related indicators.^34^ Through investigation, it was observed that ALT, AST, TG, and TC levels in the mice serum were markedly ascending, and mice liver tissues were severely damaged following HFD treatment. Subsequent interference with Asprosin diminished ALT, AST, TG, and TC levels and pathological injury of mice liver tissues in NAFLD mice stimulated by HFD.

Inflammation and lipid signaling are intertwined modulators of homeostasis and immunity.^35^ As reported, the crosstalk between metabolism, microbes, and immunity and their interplay is associated with the pathogenesis of NAFLD.^36^ Lipid deposition in the liver caused by lipid metabolism disorder is deemed as the direct cause of NAFLD.^37^ As reported, Asprosin overexpression potentiates lipid deposition.^38^ The present work also illuminated that the increased mice liver weight, the obvious hepatic lipid deposition, and the elevated TG and TC levels in the liver tissues of HFD‐induced NAFLD mice were all reduced following the absence of Asprosin. Similarly, Asprosin silencing abolished the raised TG level and the apparent lipid accumulation in PA‐exposed AML‐12 cells. HMGCR is a rate‐limiting enzyme of cholesterol synthesis in livers.^39^ FABP1 is highly expressed in hepatocytes involved in fatty acid uptake and lipid synthesis.^40^ FAS and CPT1A are pivotal enzymes responsible for lipogenesis and fatty acid oxidation.^41^ The existing experimental results disclosed that HFD exposure and PA treatment, respectively resulted in the increase in HMGCR, FABP1, and FAS expression and the decrease in CPT1A expression in the liver tissues of NAFLD mice and AML‐12 cells, which were all restored by downregulation of Asprosin.

In particular, the immune system plays an integral role. Triggers for inflammation rooted in hepatic and extrahepatic systems may lead to unique immune‐mediated pathomechanisms in NAFLD.^42^ As an initiating factor for NAFLD, aberrant hepatocyte lipid metabolism also contributes to the release of proinflammatory cytokines and inflammatory cell infiltration, further aggravating liver tissue injury.^43^ Furthermore, the proinflammatory effects of Asprosin in a variety of tissues has been reported.^44^ For instance, Asprosin has been clarified to exacerbate inflammatory response in pancreatic β‐cells.^45^ TNF‐α, IL‐1β, and IL‐6 regulated by NF‐kappaB p65 are classical proinflammatory mediators related to the development of NAFLD.^46^ As expected, the augmented TNF‐α, IL‐1β, IL‐6, and p‐p65 expression in the liver tissues of NAFLD mice caused by HFD treatment or in AML‐12 cells challenged with PA were all declined by Asprosin deficiency. All these findings pointed out that Asprosin insufficiency alleviated inflammatory response during the process of NAFLD. Hence, it was speculated that Asprosin might be a target candidate for dysregulation of hepatic immunity in the progression of NAFLD, which might also imply the potential therapeutic value of Asprosin in inflammatory and immune disorders.

AMPK, a key regulator of energy metabolism contributing to the energy of cell homeostasis, has been proposed as a potential therapeutic target of NAFLD.^18^ For instance, AMPK signaling may alter in NAFLD dependent on androgen receptor signaling, which is linked to immune response.^47^, ^48^ In healthy hepatocytes, AMPK controls the activity of enzymes involved in lipogenesis through phosphorylation and dephosphorylation.^49^ Here, HFD or PA treatment lowered p‐AMPK expression while increased p‐p38 expression in mice liver tissues or AML‐12 cells, which were reversed when Asprosin was downregulated, suggesting that inhibition of Asprosin might activate the AMPK‐p38 signaling in NAFLD. The addition of AMPK inhibitor Compound C further offset the impacts of Asprosin absence on AMPK‐p38 signaling, lipid deposition as well as inflammatory response in PA‐exposed AML‐12 cells.

Also, this study has certain limitations. For example, the expression and clinical value of Asprosin in the serum and liver tissues of patients with NAFLD are needed to be further explored in the future. The impacts of Asprosin overexpression on lipid accumulation and inflammatory response in the NAFLD mice model also need to be clarified.

## 5 CONCLUSION

In all, this study expounded that Asprosin inhibition obstructed lipid accumulation and inflammatory response in NAFLD through activating AMPK signaling (Figure S2), which emphasized the significant role of Asprosin in the development of NAFLD and might provide a potential molecular target for the treatment of NAFLD.

## AUTHOR CONTRIBUTIONS

**Bo Zhang**: Conceptualization; formal analysis; writing—original draft; writing—review & editing. **Jinger Lu**: Conceptualization; data curation; writing—original draft; writing—review & editing. **Yuhua Jiang**: Methodology; software; writing—review & editing. **Yan Feng**: Conceptualization; writing—original draft; writing—review & editing.

## CONFLICT OF INTEREST STATEMENT

The authors declare that there is no conflicts of interest.

## ETHICS STATEMENT

The authors are accountable for all aspects of the work in ensuring that questions related to the accuracy or integrity of any part of the work are appropriately investigated and resolved.

## Supporting information

Figurementary Figure  1 Experimental flow chart. HFD, high‐fat diet.

Figurementary Figure  2 Schematic figure. Asprosin inhibition activates AMPK signaling to suppress lipid accumulation and inflammatory response in NAFLD. AMPK, AMP‐activated protein kinase. TNFα, Tumor necrosis factor alpha. IL‐1β, interleukin‐1beta. IL‐6, interleukin‐6. HMGCR, 3‐hydroxy‐3‐methylglutaryl‐coA reductase. FABP1, fatty acid binding protein‐1. FAS, fatty acid synthase. CPT1A, carnitine palmitoyltransferase 1 A.

## Data Availability

All data included in this study are available upon request through contact with the corresponding author.
