# Supplementary figures and images for: Asprosin contributes to nonalcoholic fatty liver disease through regulating lipid accumulation and inflammatory response via AMPK signaling
Source: Immun Inflamm Dis. 2023 Aug 18;11(8):e947. doi: 10.1002/iid3.947 (PMC10436697; doi:10.1002/iid3.947)

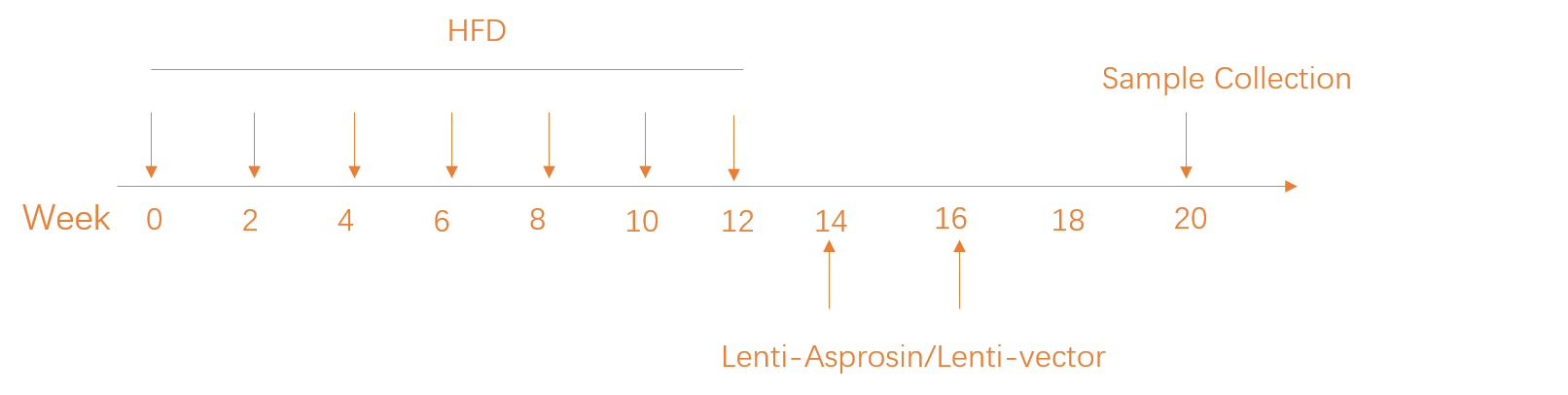

Supplement: Supplementary file 1 — Figurementary Figure 1 Experimental flow chart. HFD, high‐fat diet. [file IID3-11-e947-s002.tif]

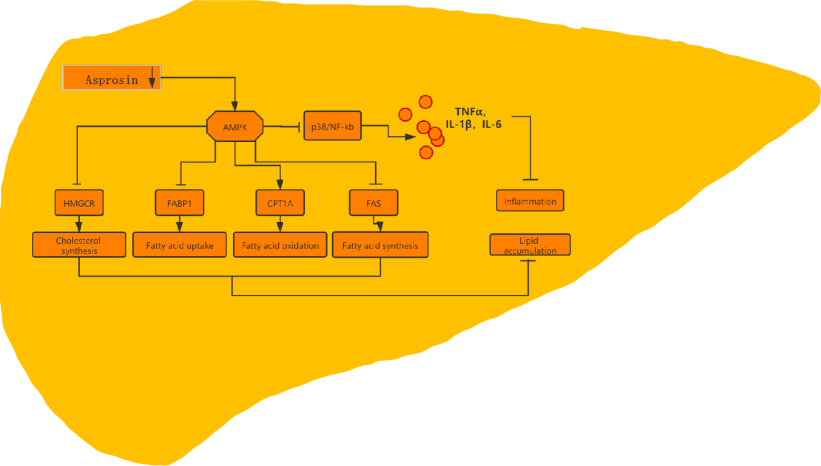

Supplement: Supplementary file 2 — Figurementary Figure 2 Schematic figure. Asprosin inhibition activates AMPK signaling to suppress lipid accumulation and inflammatory response in NAFLD. AMPK, AMP‐activated protein kinase. TNFα, Tumor necrosis factor alpha. IL‐1β, interleukin‐1beta. IL‐6, interleukin‐6. HMGCR, 3‐hydroxy‐3‐methylglutaryl‐coA reductase. FABP1, fatty acid binding protein‐1. FAS, fatty acid synthase. CPT1A, carnitine palmitoyltransferase 1 A. [file IID3-11-e947-s001.tif]
